# 24-nt-phasiRNA associated CHG/CHH DNA methylation contributes to growth heterosis of *Populus deltoides*

**DOI:** 10.48130/forres-0026-0021

**Published:** 2026-06-24

**Authors:** Jing Zhang, Weixi Zhang, Changjun Ding, Qi Liu, Zhengsai Yuan, Lulan Miao, Yanguang Chu, Xiaohua Su

**Affiliations:** 1 State Key Laboratory of Tree Genetics and Breeding, Research Institute of Forestry, Chinese Academy of Forestry, Beijing 100091, China; 2 Binhai Forestry Research Center of National Forestry and Grassland Administration, Research Institute of Forestry, Chinese Academy of Forestry, Beijing 100091, China; 3 Co‐Innovation Center for Sustainable Forestry in Southern China, Nanjing Forestry University, Nanjing 210037, China; 4 Rice Research Institute, Guangdong Academy of Agricultural Science, Guangzhou 510640, China

**Keywords:** 24-nt phasiRNA, DNA methylation, Growth heterosis, Poplar, Epigenetic, siRNA

## Abstract

24-nt siRNA-directed DNA methylation is crucial for heterosis in herbaceous plants, but its regulatory function, particularly 24-nt phasiRNA (phased small RNA)-mediated DNA methylation, remains unexplored. This study analyzed whole-genome DNA methylation and small RNA expression between high-growth (H2) and low-growth (L3) *Populus deltoides* hybrids and their parents, investigating how 24-nt phasiRNA-associated DNA methylation contributes to heterosis. Methylation levels in hybrids and parents ranged from 11.30% to 12.30%, with 22.27% to 23.34% of methylation sites being hybrid-specific. Differentially methylated regions (DMRs) between hybrids and parents mainly originated from similarly methylated regions in parents. Hypo-DMRs were more prevalent in H2, which was the opposite in L3. In the promoter region, expression of differentially methylated genes was correlated with DMR methylation levels between hybrids and parents, albeit exhibiting opposite trends in H2 and L3. Approximately 9% of 24-nt phasiRNAs in hybrids showed additive, dominant, or over-dominant inheritance, with H2 showing paternal bias and L3 maternal bias. 24-nt phasiRNAs were positively correlated with promoter CHG/CHH-DMR methylation levels and negatively correlated with non-additive gene expression, which may facilitate heterosis formation. Gene expression associated with 24-nt phasiRNAs-linked DNA methylation in hybrids was distinctly different, with H2 mainly showing high 24-nt phasiRNA expression, hyper-DMRs, and decreased gene expression, while L3 mainly showed low 24-nt phasiRNA expression, hypo-DMRs, and increased gene expression. We identified 13 candidate 24-nt phasiRNAs and seven key genes, specifically involved in nitrogen response (*NLP2/NLP8-like*) and photosynthesis (*PsbP*), which may play an important role in heterosis. This study provides the first evidence of 24-nt phasiRNA-associated DNA methylation in tree heterosis, offering novel insights into epigenetic modifications.

## Introduction

Hybrid vigor, also known as heterosis, represents a profound genetic phenomenon characterized by the superior performance of F1 hybrids resulting from crosses between genetically diverse parental lines. This enhanced performance is reflected in multiple traits, including vitality, growth potential, physiological metabolism, stress tolerance, and yield, thereby surpassing both parental lines^[1]^. Heterosis is a cornerstone of modern plant breeding, widely exploited in hybrid breeding programs of crops and forest species, including *Oryza sativa*^[2]^, *Zea mays*^[3]^, *Brassica napus*^[4]^, *Populus*^[5]^, and *Eucalyptus*^[6]^. Its application has yielded substantial economic benefits in agriculture and forestry, as well as significant social and ecological benefits^[7]^.

The mechanisms driving heterosis are complex, with ecological, physiological, and morphological traits intricately linked to its expression^[5]^. Advancements in high-throughput sequencing and bioinformatics have enabled researchers to build on classical genetic theories, such as allelic dominance and overdominance, to elucidate the molecular basis of heterosis through multi-omics analyses^[8,9]^. Evidence suggests that allele-specific expression and non-additive differential expression in F1 hybrids are key drivers of heterosis, particularly for traits like plant architecture and seed yield in crops including rice, maize, and tomato^[2,10−14]^. Non-additive genetic effects, including dominant and overdominant expression patterns, markedly enhance biomass heterosis in species such as poplar and rubber trees^[15,16]^. Additionally, epigenetic modifications—particularly DNA methylation and small RNA—play pivotal roles in transgenerational gene regulation and phenotypic variation^[4,10,17,18]^. These genetic and epigenetic insights provide critical theoretical support for breeding strategies aimed at improving productivity and resilience in agriculture and forestry.

DNA methylation plays a pivotal role in plant development, encompassing genomic stability, genomic imprinting, gene variation, and gene regulation, and is also crucial for heterosis. In species such as *Arabidopsis thaliana*, *B. napus*, and *Cajanus cajan*, DNA methylation levels in heterotic hybrids are typically higher than in their parental lines^[4,12,19]^. However, among forest tree species, hybrids exhibit significant variation in DNA methylation levels relative to their parents. For example, growth-superior hybrids of *Populus deltoides* exhibit elevated DNA methylation levels^[15]^, while hybrids of *Larix* and *Abies* exhibit hypomethylation, predominantly through negative regulation of gene expression, thereby contributing to heterosis.

Interactions between distinct parental DNA methylation states during Mendelian inheritance can generate non-additive epiallele expression and DNA methylation in hybrids^[19,20]^. These non-additive epigenetic effects are critical for heterosis, particularly for biomass traits in *Arabidopsis* hybrids^[21]^. In super hybrid rice, parental CHG methylation differences modulate allele-specific methylation and expression^[2]^. Differentially methylated regions (DMRs) between parents can contribute to methylation remodeling in hybrids and may affect non-additive methylation and gene expression, thereby influencing heterosis-related gene regulatory networks^[20,21]^.

Small RNAs, inclusive of small interfering RNAs (siRNAs) and microRNAs (miRNAs), affect gene expression through post-transcriptional mechanisms and epigenetic modifications^[22]^. In plants, the RNA-directed DNA methylation (RdDM) pathway, which is mainly mediated by 24-nucleotide (24-nt) siRNAs, plays key roles in *de novo* methylation, heterochromatin maintenance, transposon silencing, and transcriptional regulation^[7,23−26]^. Variations in siRNA abundance, alongside the expression levels of genes associated with their biogenesis, can alter genomic DNA methylation and the expression of related genes^[27,28]^. Research has shown that 24-nt siRNA-directed DNA methylation is essential for trait heterosis in various plant species, such as *Arabidopsis*, maize, rice, rapeseed, and *Cicer arietinum*. The non-additive expression of 24-nt siRNAs is linked to DMRs between hybrids and parents, influencing the non-additive differential expression of transcription factors and hormone-related genes (e.g., auxin and salicylic acid)^[4,12,19,29]^. This regulatory mechanism impacts growth and defense processes, contributing to hybrid vigor.

Phased siRNAs (phasiRNAs) are secondary small interfering RNAs with a distinct biogenetic pathway, triggered by miRNA-mediated cleavage and processed by Dicer-like proteins. Existing studies have suggested that phasiRNAs may play a guiding role in DNA methylation modifications; however, this regulatory relationship has been reported only in gramineous plants^[30,31]^. For example, 24-nt phasiRNAs biogenesis depends on RDR6 and affects CHH methylation levels during meiosis in the rice *Osrdr6-mei* mutant^[32]^. Furthermore, 24-nt phasiRNAs may *cis*-regulate CHH methylation levels at *24-PHAS* loci and adjacent regions, and their absence causes male sterility and reduced DNA methylation in maize^[31]^. Overall, phasiRNA involvement in DNA methylation has not been reported in woody plants, and the specific mechanisms by which 24-nt phasiRNA-mediated DNA methylation contributes to heterosis remain to be further elucidated.

Heterosis holds great potential for enhancing forestry productivity, as demonstrated by successful poplar hybrid breeding^[33,34]^. However, poplar's intricate genetic landscape and long growth cycles complicate the molecular understanding of heterosis^[15]^. This study aims to dissect the genetic and epigenetic mechanisms governing heterosis in *P. deltoides* by analyzing intraspecific F1 hybrids with varying growth vigor. Using whole-genome bisulfite sequencing (WGBS) and transcriptome sequencing, we analyzed the expression profiles of 24-nt phasiRNAs, DNA methylation patterns, and associated regulatory networks. Our results offer novel perspectives and a theoretical foundation for epigenetic mechanisms underlying forest heterosis, as well as their application in precision hybrid breeding.

## Materials and methods

### Plant materials

This study used high-growth F1 hybrid (H2) and low-growth F1 hybrid (L3) of *Populus deltoides*, along with their male parent (*P. deltoides* cl. '10/17', MP) and female parent (*P. deltoides* cv. '55/65', FP) as experimental materials, which were previously screened by our research group^[15,35]^. The female parent (FP) exhibited greater tree height than the male parent (MP) at both 1 and 3 years of age, and subsequently maintained this growth advantage in tree height^[15,36]^. The experimental forest was constructed by uniform cuttings taken from newly germinated branches in 2019, in Xunfeng Town, Xiuwu County, Jiaozuo City, Henan Province^[37]^. Sample collection and measurement of tree height and diameter at breast height (DBH) were conducted in three test blocks in August 2021. In each block, six trees with healthy and uniform growth were chosen per test line (six trees × four lines = 24 total) to measure tree height and DBH using the Vertex IV instrument (Haglof, Sweden) and diameter tape, respectively. Of which, three trees with consistent growth were selected per test line, and the fourth to sixth mature leaves from the east, south, west, and north branches at 2/3 of the tree height were collected at 9:00 AM on a fine day. These leaves were immediately frozen in liquid nitrogen and stored at −80 °C for omics sequencing (12 samples in total).

### Whole genome bisulfite sequencing and analysis

Genomic DNA was extracted from leaves using the cetyltrimethylammonium bromide (CTAB) method. Libraries were performed for each line in three biological replicates. DNA was fragmented to 200−300 bp by ultrasonication, followed by end repair, 3' A base addition, and adapter ligation. Bisulfite treatment was performed using an EZ DNA Methylation-Gold Kit (Zymo Research, USA). Libraries were purified, size-selected, and PCR-amplified before whole-genome bisulfite sequencing (WGBS) on the MGISEQ2000 platform (PE150 model) at BGI (Shenzhen, China). Raw data from WGBS were filtered using SOAPnuke software^[38]^, and clean reads were aligned to the *P. deltoides* reference genome (https://phytozome-next.jgi.doe.gov/info/PdeltoidesWV94_v2_1) using Bismark v0.22.1 software^[39]^.

DNA methylation levels were defined as the proportion of methylated reads relative to all reads covering a given site^[40]^. The calculation formula is as follows:

1 $ {\mathrm{Rm}}_{\mathrm{average}}=\frac{{\mathrm{Nm}}_{\mathrm{all}}}{{\mathrm{Nm}}_{\mathrm{all}}+{\mathrm{Nnm}}_{\mathrm{all}}}\times 100{\%} $

Nm represents the number of reads supporting the methylated cytosine at a given site, while Nnm denotes the number of reads supporting the unmethylated cytosine at that site.

The entire genome was divided into 200 bp bins for the identification of differentially methylated regions (DMRs). The R package DMRcaller was used to calculate DMRs between samples or between groups^[41]^. Only bins that contained a minimum of five cytosines, with each cytosine covered by at least five reads, were retained for further analysis. DMRs were identified in each 200 bp bin with the following parameters: (1) mean methylation difference > 30% for mCG, > 20% for mCHG, and > 10% for mCHH; (2) DMRs with a group-wise Q-value (adjusted *p*-value) < 0.05. Regions between parents not meeting DMR criteria were classified as parental similarly methylated regions (SMRs). DMRs with higher methylation were termed Hyper-DMRs, while those with lower methylation were termed Hypo-DMRs. All methylation analyses were conducted using the Dr. Tom online platform (https://biosys.bgi.com). Using BEDTools, DMR-associated genes (DMGs) and other genomic features were annotated according to genomic coordinates^[42]^. A DMR was considered associated with a feature when the overlap exceeded 1 bp. DMRs were classified as upstream 3 kb, gene body, downstream 3 kb, transposable element (TE), or intergenic region. When a DMR overlapped multiple features, annotation followed the priority order: upstream 3 kb > gene body > downstream 3 kb > TE > intergenic.

### RNA sequencing and analysis

Total RNA was extracted using the CTAB method. Small RNA sequencing libraries were constructed for each line with three biological replicates, followed by sequencing on the DNBSEQ platform (SE50 mode) at BGI (Shenzhen, China). Raw data were processed to remove adapter contamination, low-quality reads, and tRNA/rRNA sequences using SOAPnuke (1.5.0) software^[38]^. Clean reads were aligned to *P. deltoides* reference genome using Bowtie (v1.1.2) software^[43]^. High-quality sequences were annotated, and the 24-nt phasiRNAs annotated in the plant non-coding RNA database were summarized. Expression levels were normalized using TPM (Transcripts Per Million) and quantified. Differential expression analysis of 24-nt phasiRNAs between groups was performed with DESeq2 software^[44]^, with a threshold of |log_2_Fold Change| ≥ 0.5 and *p-*value ≤ 0.05. Non-additive 24-nt phasiRNAs were identified with |Log_2_(F1 vs MPV)| ≥ 0.5 and *p-*value ≤ 0.05. Additive 24-nt phasiRNAs were identified with no significant difference between the F_1_ hybrid and MPV. Conserved 24-nt phasiRNAs were identified with no significant differences between the F_1_ hybrid and its parents, as well as between the two parents.

Previously published data were used for gene expression analysis^[37]^. Gene Ontology (GO) and Kyoto Encyclopedia of Genes and Genomes (KEGG)^[45]^ enrichment analysis were carried out to identify genes related to growth using the Dr. Tom tool (www.biosys.bgi.com).

### Correlation analysis of 24-nt phasiRNA, DMR methylation, and gene expression

When identifying differentially methylated genes (DMGs) from WGBS data, genes with expression level met the threshold of |Log_2_FoldChange| ≥ 1 and FDR ≤ 0.05 were classified as DMG-DEGs. To identify potential networks in which 24-nt phasiRNA-mediated methylation regulates gene expression, matching and localization were performed using the genomic coordinates of 24-nt phasiRNAs and DMRs between hybrids and parents. For the matched 24-nt phasiRNA-DMR-gene pairs, correlation analysis was conducted between 24-nt phasiRNA expression and DMR methylation levels, and between DMR methylation levels and gene expression levels. A correlation coefficient ≥ 0.70 or a *p*-value ≤ 0.05 was considered statistically significant. Considering the in-depth analysis of sRNA-seq, WGBS, and RNA-seq data, the number of genes gradually decreased, and whole transcriptome analysis may influence the identification and selection of both sRNAs and genes. Therefore, genes influenced by 24-nt phasiRNA, with expression thresholds of Fold Change ≥ 1.5 and FDR ≤ 0.05 were designated as 24-nt phasiRNA-DMR-DEGs. Correlation analysis and heatmap visualizations were performed using OmicShare tools (www.omicshare.com/tools).

### QRT-PCR validation

To confirm the reliability of the RNA-seq data, 10 genes were chosen for qRT-PCR validation. Leaf tissues were ground in liquid nitrogen, and total RNA was isolated using the RNAprep Pure Plant Kit (Tiangen, Beijing, China). qRT-PCR was performed using a LightCycler 480 II Real Time PCR Instrument (Roche, Switzerland), and analyzed with SYBR Premix Ex Taq II (Takara, Japan) according to the manufacturer's protocol^[37]^. The assay was repeated three times per sample after mixing. *Actin* (Gene ID: OX637672.1) served as the internal reference gene. Gene-specific primers were designed (Supplementary Table S1), and the relative expression of genes was calculated using the 2^−ΔΔCᴛ^ method^[46]^.

### Statistical analysis

Heterosis values for tree height and DBH were calculated using these formulas^[37]^:

2 $ \text{Mid-parent heterosis}\left(\mathrm{MPH}\right)=\frac{{\mathrm{F1}}-{\mathrm{MPV}}}{\mathrm{MPV}}\times 100{\%} $

3 $ \text{High-parent heterosis}\left(\mathrm{HPH}\right)=\frac{{\mathrm{F1}}-{\mathrm{HP}}}{\mathrm{HP}}\times 100{\%} $

The average trait value of both parents, referred to as the mid-parental value (MPV), and the trait value of the higher parent (HP) were used in the analysis.

Tree height and DBH data (means ± SD) were analyzed by using One-way analysis of variance (One-way ANOVA) with SPSS 25.0 software at the significance level of *p*-value < 0.05 (*). Graphical presentation of the data was generated using Origin 2021, and image layouts were prepared using Adobe Illustrator 2020.

## Results

### DNA methylation divergence between hybrids and parents

The study utilized the female parent (FP, *P. deltoides* cv. '55/65'), male parent (MP, *P. deltoides* cl. '10/17'), and their F1 hybrids. We employed a completely randomized block design to establish a clonal test plantation. Tree height and diameter at breast height (DBH) were measured for 3-year-old trees. The high-growth hybrid (H2) exhibited the highest values for both tree height and DBH, significantly surpassing the parental lines (*p* < 0.05) (Supplementary Table S2). In contrast, the low-growth hybrid (L3) demonstrated the lowest values. Specifically, H2 showed mid-parent heterosis (MPH) of 9.98% and 5.21% for tree height and DBH, respectively, and high-parent heterosis (HPH) of 9.16% and 2.75% (Supplementary Table S2). Conversely, L3 showed MPH of −8.28% and −19.22%, and HPH of −8.96% and −21.11% for tree height and DBH, respectively. Based on these findings, we investigated DNA methylation divergence between hybrids (H2 and L3) and parents (FP and MP).

**Figure 1:**
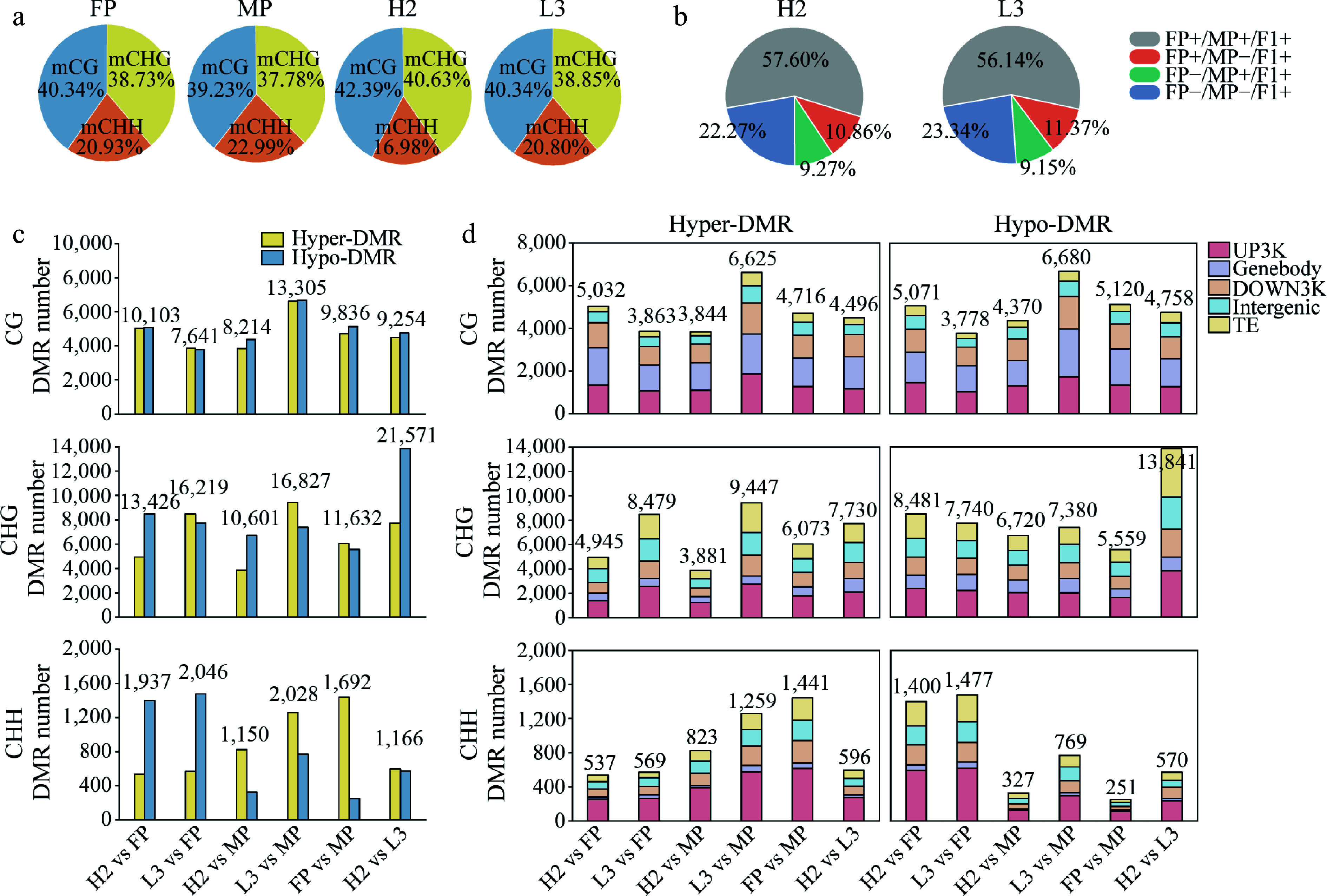
Distribution of genome-wide methylation and differentially methylated regions (DMRs) among genomic regions in parents and hybrids. (a) Percentage of reads mapping to different methylation sites. (b) Contribution of parental methylation sites to hybrid methylation sites. (c) Number of hyper-DMRs (hypermethylated regions) and hypo-DMRs (hypomethylated regions) in different comparison groups. (d) Distribution of hyper- and hypo-DMRs across various genomic regions in different comparison groups. UP3Kb: gene upstream 3Kb; Genebody: gene body; DOWN3Kb: gene downstream 3Kb; Intergenic: intergenic region; TE: transposable element.

WGBS yielded 168 Gb of clean bases, with mapping rates across all libraries ranging from 67.78% to 72.81%. Unique mapping rates ranged from 53.17% to 56.66%, and bisulfite conversion rates varied from 99.37% to 99.48% (Supplementary Table S3). Whole-genome methylation levels were analyzed, and a global methylation map was drawn (Supplementary Fig. S1a). At cytosine (C) sites, the methylation level in L3 (12.30%) was similar to that of FP (12.08%), whereas H2 (11.65%) was similar to MP (11.30%); notably, L3 and FP exhibited higher overall methylation than H2 and MP (Supplementary Fig. S1b). CG sites showed the highest methylation levels, followed by CHG sites (Supplementary Fig. S1c−S1e). Compared to parents and L3, H2 showed a distinct methylation profile, with a higher proportion of CG methylation (mCG) and mCHG, and a lower proportion of mCHH (Fig. 1a).

Further analysis showed that 77.73% and 76.66% of methylation sites in H2 and L3, respectively, were shared with their parents. Particularly, 20.13% and 20.52% of methylation sites in H2 and L3, respectively, exhibited parent-specific characteristics, whereas 22.27% and 23.34% of methylation sites were hybrid specific (Fig. 1b). The number of differentially methylated regions (DMRs) between H2 and parents was lower than that between L3 and parents (Fig. 1c). Moreover, the number of DMRs between H2 and FP (25,466) was higher than that between H2 and MP (19,965), whereas L3 exhibited the opposite trend. H2 exhibited more hypomethylated sites, while L3 had a higher prevalence of hypermethylated sites. These results highlight significant differences in methylation patterns between hybrids with different growth vigors. Notably, the DNA methylome of the F1 hybrids exhibited clear parental bias, with H2 more closely resembling the male parent and L3 more closely resembling the female parent, consistent with their gene expression patterns^[37]^.

The distribution of DMRs between hybrids and parents across genomic features (UP3Kb, genebody, DOWN3Kb, intergenic regions) and transposable elements (TEs) was analyzed (Fig. 1d). CG DMRs were predominantly located within genebody and UP3Kb regions, whereas CHG and CHH DMRs were most abundant in the UP3Kb region and least frequent in the genebody region. Overall, CG, CHG, and CHH DMRs exhibited high enrichment in the UP3Kb region, suggesting that these DMRs are primarily localized to regions associated with transcriptional regulation.

### Identification of differentially expressed genes in DMRs

**Figure 2:**
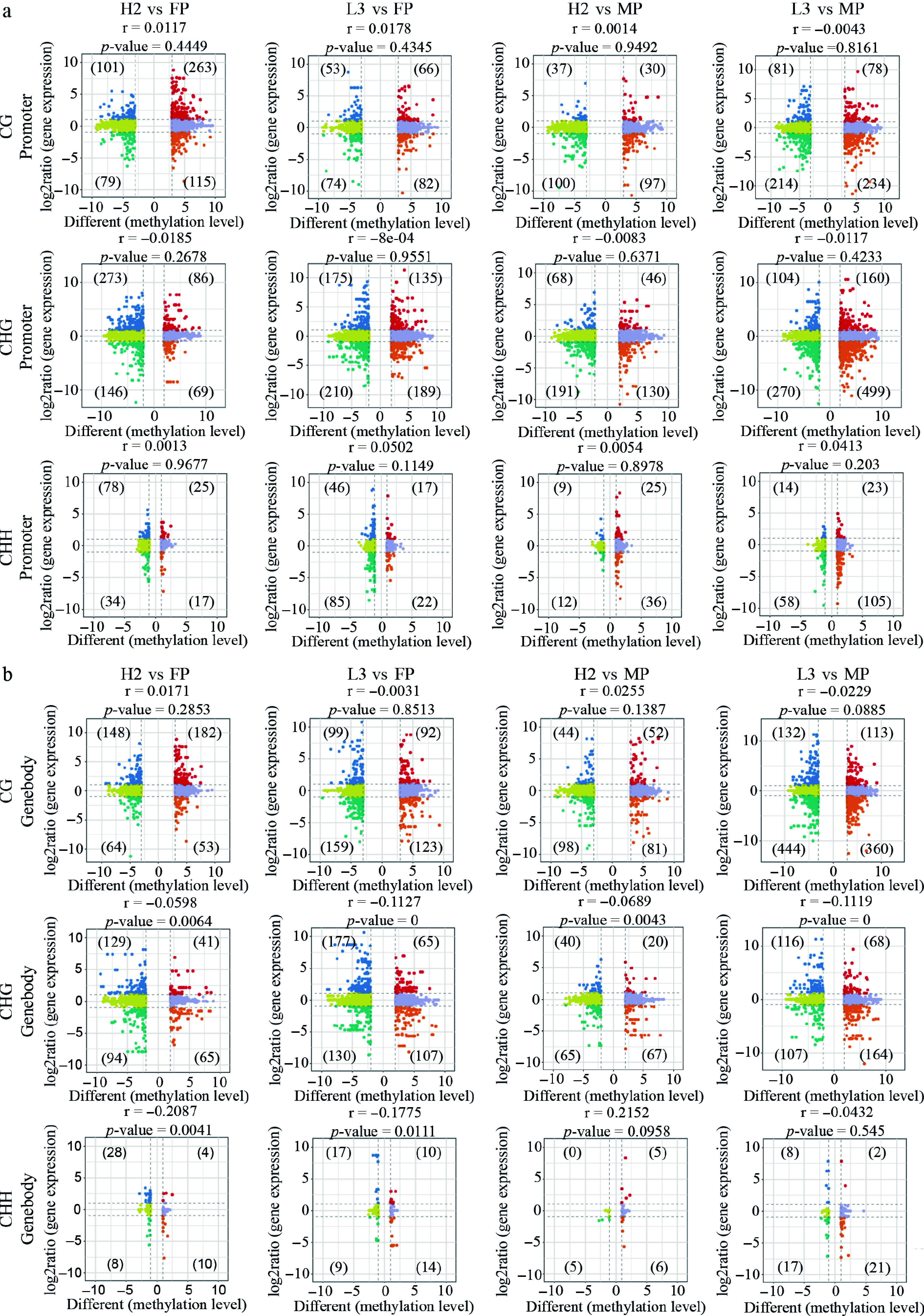
Correlation between gene expression and methylation levels of differentially methylated regions (DMRs) between hybrids and parents in parental similarly methylated regions (SMRs). (a) Nine-quadrant plot illustrating the relationship between gene expression and DMR methylation levels in the promoter region. (b) Nine-quadrant plot displaying the correlation between gene expression and DMR methylation levels in the genebody region.

**Figure 3:**
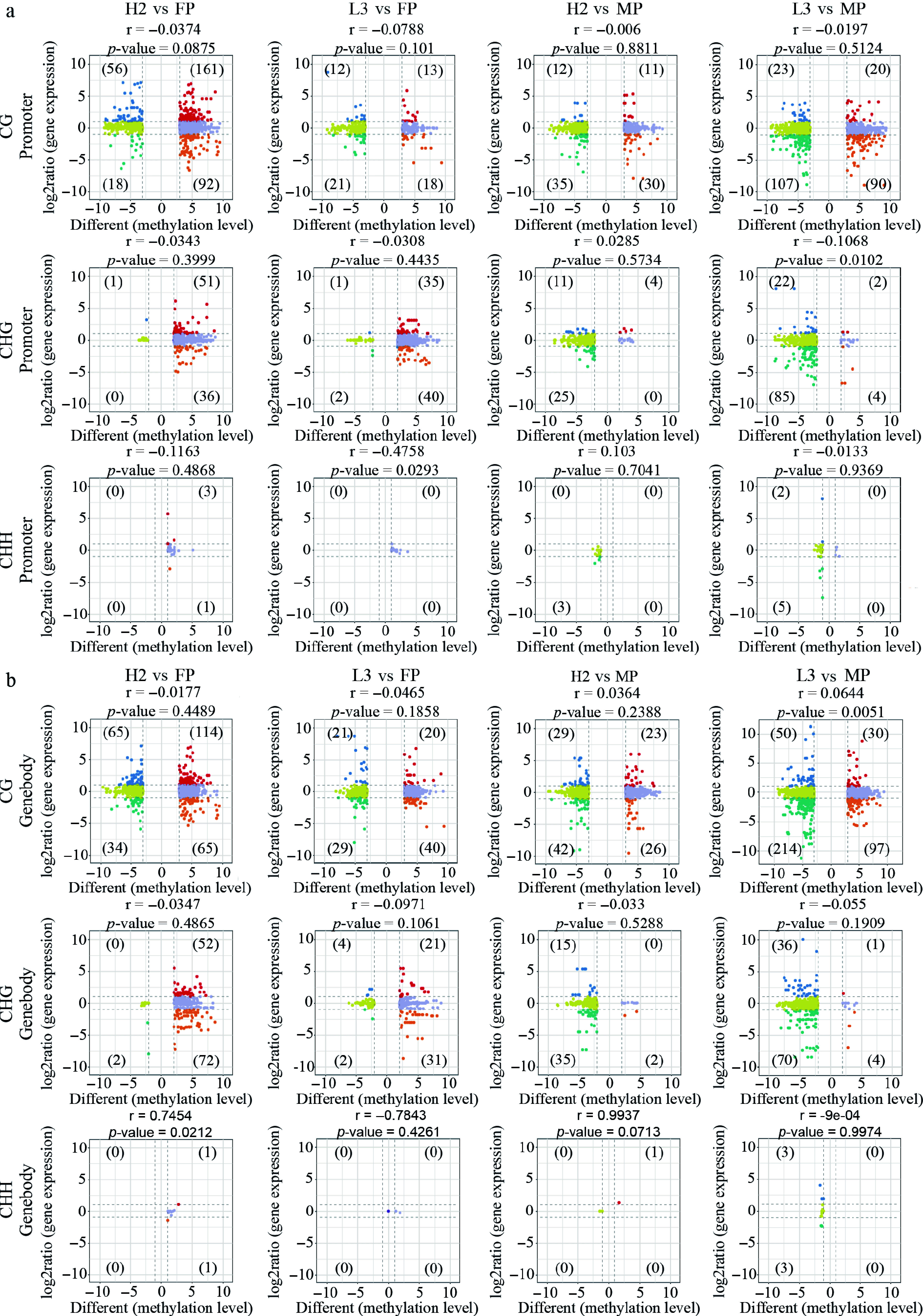
Correlation between gene expression and methylation level of differentially methylated regions (DMRs) between hybrids and parents in parental DMRs. (a) Nine-quadrant plot illustrating the relationship between gene expression and DMR methylation levels in the promoter region. (b) Nine-quadrant plot displaying the correlation between gene expression and DMR methylation levels in the genebody region.

DMRs are predominantly found in the upstream 3 kb (UP3Kb) and genebody regions, suggesting that methylation differences may influence gene expression. To investigate the impact of parental DNA methylation on hybrids' gene expression, DMRs between hybrids and parents were categorized into parental similarly methylated regions (SMRs) and parental DMRs. Correlation analysis was conducted between gene expression and methylation levels of DMRs (both parental DMRs and SMRs) in hybrids and parents. Differentially expressed genes (DEGs) with a significant correlation with DMRs were defined as differentially expressed and methylated genes (DMEGs). In total, 3,981 and 1,176 DMEGs were identified in parental SMRs and DMRs, respectively (Figs 2a, b, 3a, b).

These DMEGs were most prevalent in CG-type SMRs and DMRs, followed by CHG and CHH types. In DMEGs between different hybrids and parents, the regulatory pattern trend between DMR methylation levels and DEGs expression levels was mainly opposite (Figs 2a, b, 3a, b). In parental SMRs, CG-type promoter/genebody DMEGs in H2 mainly exhibited two patterns: hypermethylation with increased expression and hypomethylation with decreased expression. Conversely, L3 mainly exhibited hypermethylation with decreased expression in the promoter region and hypomethylation with decreased expression in the genebody region (Fig. 2a, b). Additionally, CHG- and CHH-type DMEGs in H2 primarily displayed hypomethylation with decreased expression and hypomethylation with increased expression; in L3, however, the DMEGs showed hypomethylation with decreased expression relative to the FP, but hypermethylation with decreased expression relative to the MP (Fig. 2a, b).

In parental DMRs, numerous CG- and CHG-type DMEGs were identified in promoter/genebody regions, while CHH-type DMEGs were basically absent (Fig. 3a, b). Relative to the FP, CG- and CHG-type DMEGs in H2 predominantly exhibited hypermethylation with increased expression, whereas L3 displayed hypermethylation with decreased expression. Relative to the MP, both hybrids primarily exhibited hypomethylation with decreased expression (Fig. 3a, b). Interestingly, in both parental SMR and DMR regions, DMEGs between hybrids and parents were enriched in similar functional pathways, including plant–pathogen interaction, MAPK signaling pathway-plant, and starch and sucrose metabolism (Supplementary Fig. S2a−S2c). These DMEGs were also primarily annotated under similar Gene Ontology (GO) terms (Supplementary Fig. S2b−S2d). Further analysis was conducted on all DMEGs between hybrids and parents.

### Differential expression of siRNAs across generations

**Figure 4:**
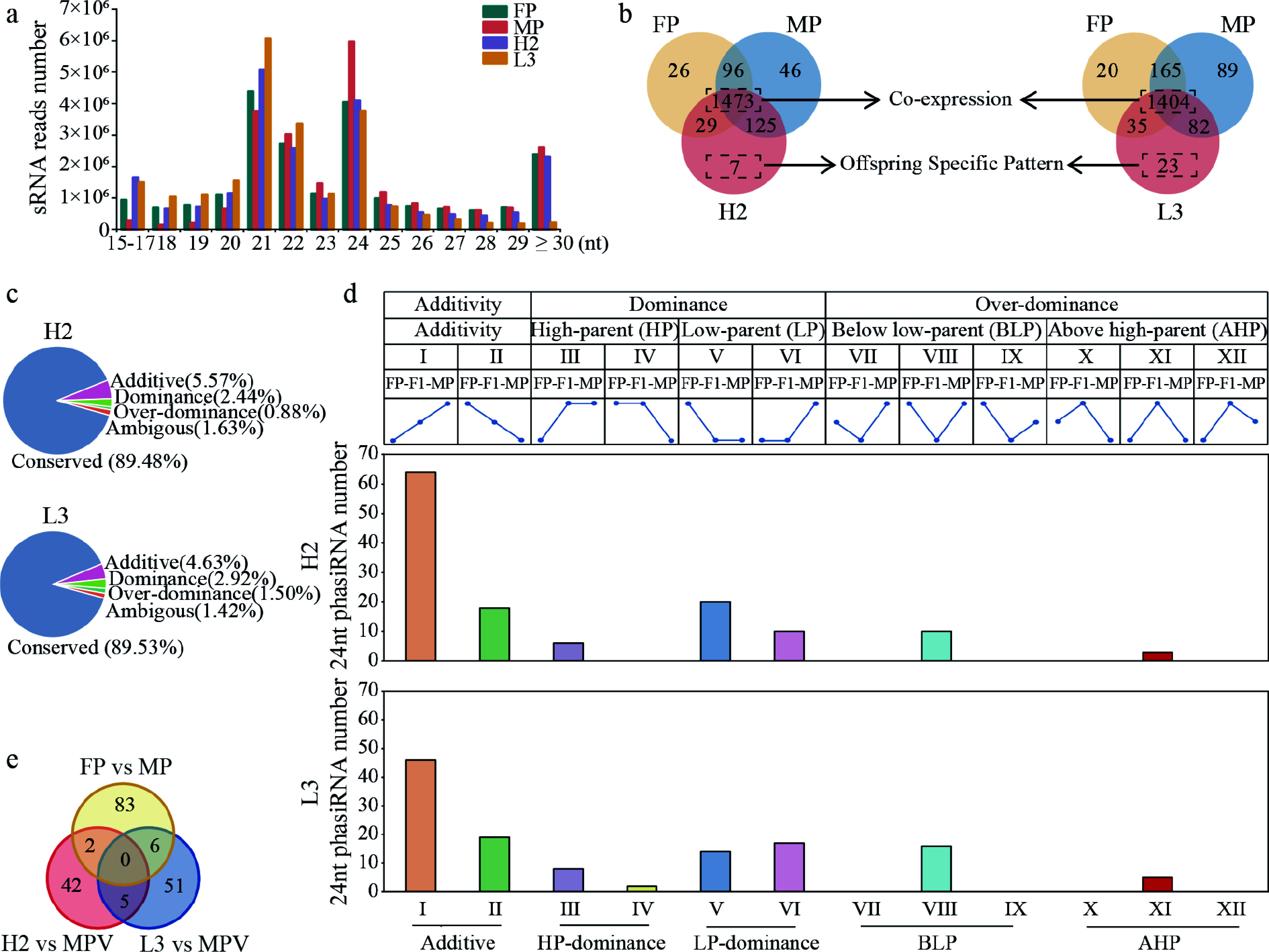
Overview of sRNA distribution and 24-nt phasiRNA expression patterns in F1 hybrids and parents. (a) Distribution of sRNA reads across lengths in parents and hybrids. (b) Venn diagram of 24-nt phasiRNAs expressed in hybrids and parents. (c) Inheritance pattern of 24-nt phasiRNAs in F1 hybrids. 'Conserved Expression' refers to expression patterns with no significant differences between parents, or hybrids and parents. Other patterns include additive, dominance, and overdominance expression. (d) Number of 12 expression patterns of unstable expressed 24-nt phasiRNAs in F1 hybrids. HP: expression resembling the high-parent expression levels. LP: expression resembling the low-parent expression levels. BLP: expression was significantly reduced compared to the low-expression parent (false discovery rate [FDR] < 0.05). AHP: expression significantly increased compared to high-parent (FDR < 0.05). (e) Venn diagram of non-additive 24-nt phasiRNAs in hybrids with different growth potentials vs differentially expressed 24-nt phasiRNAs among parents.

A total of 262.72 million clean reads were obtained by sRNA-seq, with 80.07%–88.32% aligning to the reference genome. After filtering, each library yielded approximately 4.13–5.55 million unique sRNA reads (Supplementary Table S4). The correlation between biological replicates was high (Supplementary Fig. S3a, S3b). The majority of sRNA reads were 21-nt and 24-nt in length (Fig. 4a). In total, 1,822 24-nt phasiRNAs were identified from 525 distinct *PHAS* loci. Notably, the number of 24-nt phasiRNAs was highest in MP (1,740), followed by H2 (1,634), FP (1,624), and L3 (1,544) (Fig. 4b). Overall, 24-nt phasiRNA abundance in hybrids was lower than the mid-parent value (MPV) (Supplementary Fig. S3c). One-thousand four hundred and seventy-three co-expressed 24-nt phasiRNAs were identified in H2 and parents, and 1,404 in L3 and parents (Fig. 4b; Supplementary Tables S5, S6). Furthermore, uniquely expressed 24-nt phasiRNAs were more numerous between hybrids and MP than between hybrids and FP. Interestingly, MP and L3 exhibited a higher number of uniquely expressed 24-nt phasiRNAs (Fig. 4b).

To further explore the differential expression of 24-nt phasiRNAs between parents and hybrids, co-expressed 24-nt phasiRNAs were categorized into Conserved, Additive, Dominance, and Overdominance patterns (Fig. 4c)^[19]^. Conserved expression accounted for 89.48% of co-expressed 24-nt phasiRNAs in H2 and 89.53% in L3, suggesting that most 24-nt phasiRNAs were stably inherited across generations. Among those non-stable inheritance patterns (Fig. 4c), we identified 82 additively expressed, 36 dominantly expressed, and 13 overdominant-expressed 24-nt phasiRNAs in H2. Similarly, L3 contained 65 additively expressed, 41 dominantly expressed, and 21 overdominantly expressed 24-nt phasiRNAs. Therefore, only 8.89%–9.05% of the co-expressed 24-nt phasiRNAs in H2 and L3 showed additive or non-additive differential expression. Significantly, the majority of non-additively differentially expressed 24-nt phasiRNAs exhibited downregulation, primarily following the low-parent dominance (LP) pattern (Fig. 4d), followed by the negative over-dominant (below low-parent, BLP) pattern (Fig. 4d). Among the non-additive 24-nt phasiRNAs, the number of paternal dominant expression (ELD-MP) of H2 (50.06%) was much higher than maternal dominant expression (ELD-FP) (20.41%), while the number of ELD-MP and ELD-FP patterns was almost equal in L3, accounting for 35.48% and 30.65%, respectively (Fig. 4d). These results indicate that the inheritance patterns of 24-nt phasiRNAs in H2 may be mainly biased toward the male parent, whereas no such parental bias existed in L3. Additionally, only two and six non-additive 24-nt phasiRNAs in H2 and L3, respectively, were differentially expressed between parents (Fig. 4e), indicating that parental differential expression may not be the primary driver of non-additive expression in the F1 hybrids.

### Regulation analysis of 24-nt phasiRNA, DNA methylation and gene expression in hybrids

**Figure 5:**
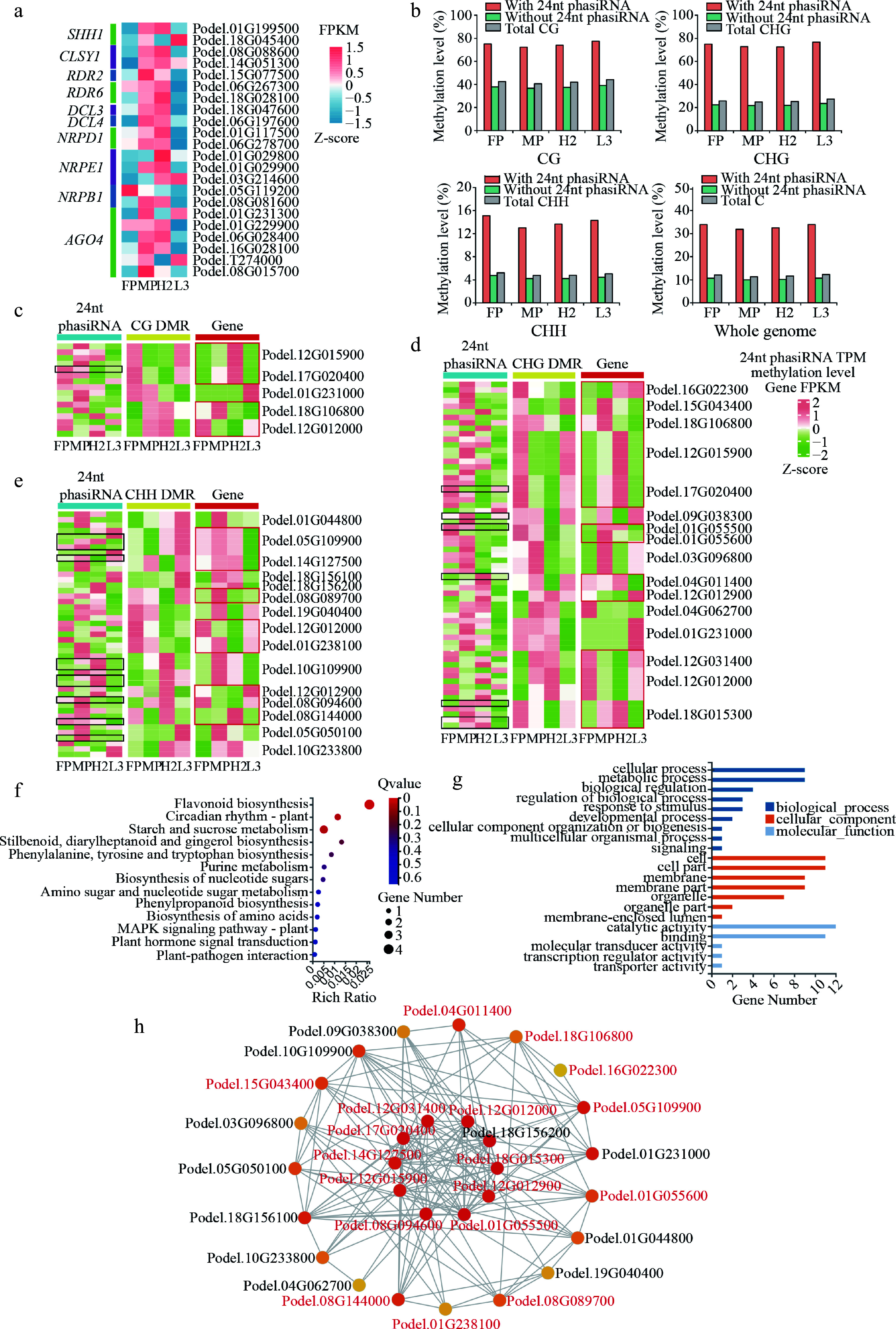
Relationship of 24-nt phasiRNA, DNA methylation, and gene expression in hybrids. (a) Heatmap of gene expression involved in 24-nt phasiRNA biosynthesis. (b) Methylation level of 24-nt phasiRNA region on chromosome. (c)−(e) Heatmaps of 24-nt phasiRNA expression levels (TPM); (c) CG, (d) CHG, and (e) CHH DMR methylation levels and differentially methylated gene expression levels (FPKM). The black box represents non-additive 24-nt phasiRNA, while the red box represents non-additive genes. (f) KEGG pathway enrichment, and (g) GO annotation of genes associated with 24-nt phasiRNA and DNA methylation. (h) Co-expression network for genes, with red-colored genes representing non-additive DEGs.

To elucidate the regulatory relationship between the biogenesis of 24-nt phasiRNAs and DNA methylation, we analyzed the expression patterns of key genes involved in the core Pol II–RDR6–DCL3/DCL5 pathway for 24-nt phasiRNA biosynthesis. A total of 22 potential key genes were identified, including *SHH1*, *CLSY1*, *RDR2*, *RDR6*, *DCL3*, *DCL4*, *NRPD1*, *NRPE1*, *NRPB1*, and *AGO4*. Expression pattern analysis revealed that the above genes mainly exhibited additive expression and high-parent dominance in H2, whereas negative overdominance and low-parent dominance were observed in L3 (Fig. 5a). In addition, at the whole-genome level, DNA methylation levels in 24-nt phasiRNA regions in both hybrids and parents were higher than in regions without 24-nt phasiRNA (Fig. 5b). Moreover, in promoter, genebody, and TE regions, 24-nt phasiRNA abundance in MP and H2 was higher than in FP and L3 (Supplementary Fig. S4a, S4b). These findings provide a biological basis for the substantial synthesis of 24-nt phasiRNAs in the high-parent hybrid H2 and are thereby correlated with DNA methylation.

To elucidate the role of 24-nt phasiRNA-associated DNA methylation in hybrids with different growth potentials, we conducted a comprehensive analysis of DMRs between hybrids and parents using genomic positions and sequence information of 24-nt phasiRNAs, followed by an examination of DEGs and non-additive DEGs across comparison groups. In parental CG-, CHG-, and CHH-type DMRs, six, seven, and two *PHAS* loci were mapped, respectively. Additionally, three, 22, and 27 *PHAS* loci were localized in the corresponding parental SMRs (Supplementary Table S7). In total, 169 24-nt phasiRNAs were identified in 65 DMRs between hybrids and parents, of which 110 24-nt phasiRNAs (65.09%) were located in DMRs of promoter, genebody, and downstream regions containing 44 expressed genes (17 DMEGs). Thirty-one out of these 44 expressed genes were in parental SMRs, a higher number than in parental DMRs. We then hypothesized that a regulatory relationship exists among these 24-nt phasiRNAs, DMRs, and genes. Further analysis revealed that, in promoter/genebody-DMR between hybrids and parents, 16 24-nt phasiRNAs (one non-additive) were associated with 5 CG-DMRs linked to eight expressed genes, 47 24-nt phasiRNAs (nine non-additive) were associated with 20 CHG-DMRs linked to 16 expressed genes, and 42 24-nt phasiRNAs (9 non-additive) with 15 CHH-DMRs related to 15 expressed genes (Supplementary Table S7). Combined analysis of 24-nt phasiRNA expression, DMR methylation, and gene expression revealed distinct association patterns in H2 and L3 (Fig. 5c−e). H2 predominantly exhibited 24-nt phasiRNAs upregulation, hypermethylation, and decreased gene expression, as well as 24-nt phasiRNAs downregulation, hypomethylation, and increased gene expression (Fig. 5c−e). In contrast, L3 more often showed 24-nt phasiRNAs downregulation, hypermethylation, and decreased gene expression in CG and CHG DMRs. Furthermore, in CHH DMRs, L3 also exhibited 24-nt phasiRNAs downregulation, hypomethylation, and increased gene expression. GO functional annotation showed that, the above 29 candidate genes linked to 24-nt phasiRNA-associated DNA methylation were primarily involved in cellular processes, metabolic processes and cells, cellular components, with an emphasis on catalytic activity and binding (Fig. 5f). These genes were primarily enriched in essential plant growth processes, including flavonoid biosynthesis, circadian rhythms, and starch and sucrose metabolism (Fig. 5f; Supplementary Table S8). Co-expression analysis of 29 genes identified nine non-additive DMEGs among 10 hub genes (Fig. 5h). Among these, five non-additive DMEGs, including *nitrogen-responsive transcription factor* (*NLP2/NLP8-like*, *Podel.17G020400*), *PSBP-like protein 1* (*Podel.12G015900*), *chalcone synthase/flavonol synthase* (*CHS/FLS*, *Podel.01G055500*), *pheophorbide a oxygenase* (*PaO*, *Podel.18G015300*), and *AIR synthase* (*Podel.14G127500*), were significantly upregulated in H2, while four genes, including *endoglucanase* (*Podel.08G094600*), and *leucine*-*rich repeat* (*LRR*, *Podel.12G012000*, *Podel.12G012900*, and *Podel.12G031400*), were downregulated (Fig. 5c−e). These genes showed opposite expression patterns in L3.

### 24-nt phasiRNA-associated CHG/CHH DNA methylation contributes to non-additive gene expression

**Figure 6:**
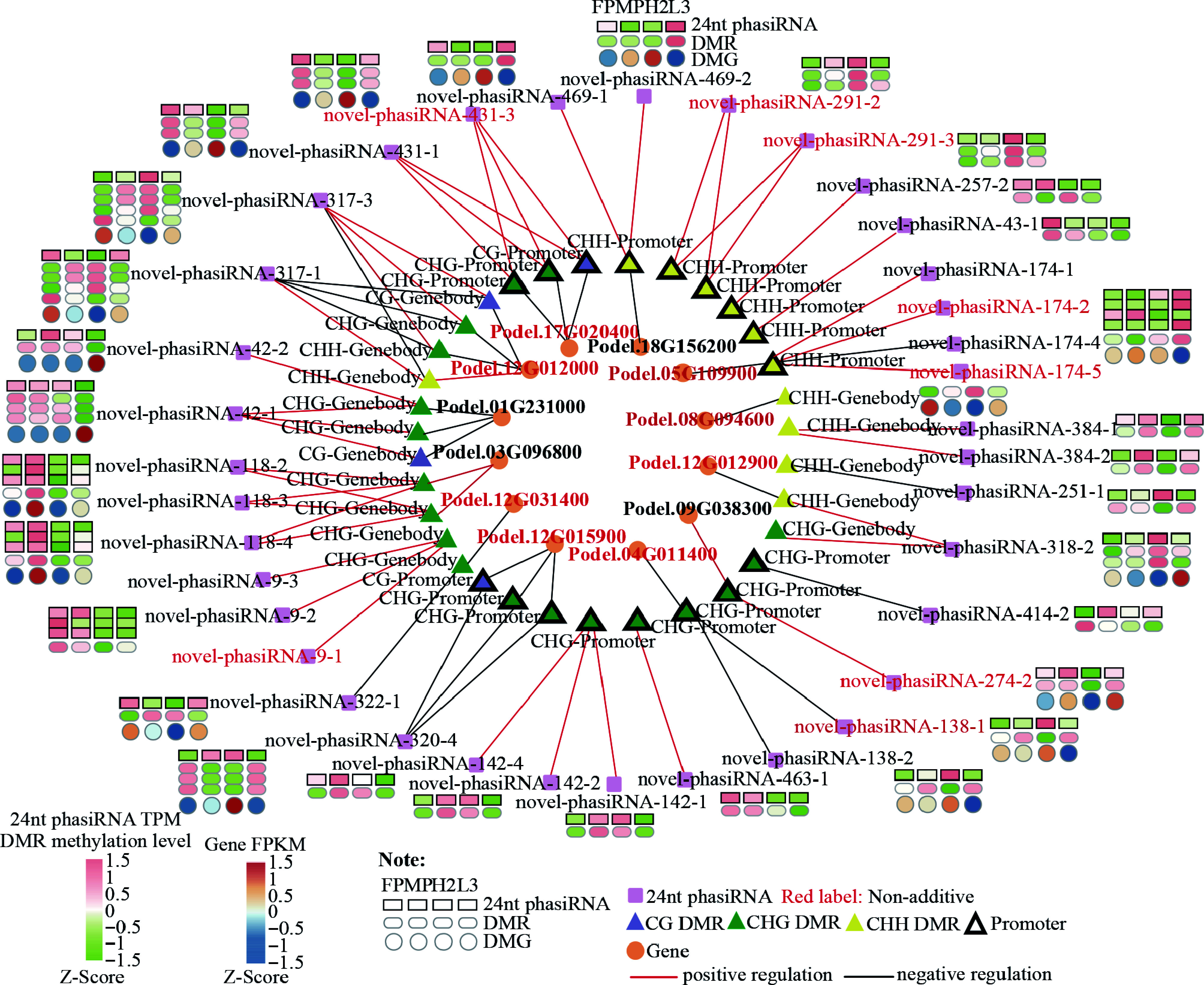
Candidate 24-nt phasiRNA-DMR-gene network showing significant associations among 24-nt phasiRNA abundance, DMR methylation, and gene expression. Red-colored genes are non-additive DEGs.

Correlation analysis was performed among 24-nt phasiRNAs, DMRs, and genes exhibiting potential regulation patterns, using a correlation coefficient ≥ 0.70 or *p*-values ≤ 0.05. A total of six 24-nt phasiRNA-CG-DMR pairs, 32 24-nt phasiRNA-CHG-DMR pairs, and 18 24-nt phasiRNA-CHH-DMR pairs were identified, primarily exhibiting positive correlations (Fig. 6). These positive correlations were mainly observed in CHH-promoter and CHG-Genebody regions. Among 22 significantly correlated DMR-gene pairs, 18 exhibited negative correlations, including nine located in promoter regions (Fig. 6). By contrast, four positively correlated DMR-gene pairs were mainly found in genebody regions. This suggests that methylation levels in upstream and transcriptional regions of genes may inhibit gene expression (Fig. 6).

Based on a significant correlation, we constructed a 24-nt phasiRNA-DMR-gene network. Within this network, 13 24-nt phasiRNAs and their associated DNA methylation were significantly correlated with seven non-additive DEGs (*Podel.05G109900*, *Podel.17G020400*, *Podel.12G012000*, *Podel.12G031400*, *Podel.12G015900*, *Podel.04G011400*, and *Podel.12G012900*). Importantly, three non-additive 24-nt phasiRNAs showed a significant negative correlation with non-additive DEGs by potentially mediating promoter-DMRs. These 24-nt phasiRNAs, along with DNA methylation and gene expression, displayed reverse patterns between H2 and L3. For instance, in H2, the non-additively downregulated novel-phasiRNA-431-3, novel-phasiRNA-174-2, and novel-phasiRNA-174-5 were associated with CHG/CHH hypomethylation and high-parent dominant gene expression (*NLP2/NLP8-like*, *Podel.17G020400*; *RRN3*, *Podel.05G109900*), whereas L3 exhibited upregulated 24-nt phasiRNAs, hypermethylation, and downregulated genes. These opposite regulation patterns might contribute to growth heterosis in *P*. *deltoides*.

### Identification of key genes for heterosis formation

**Figure 7:**
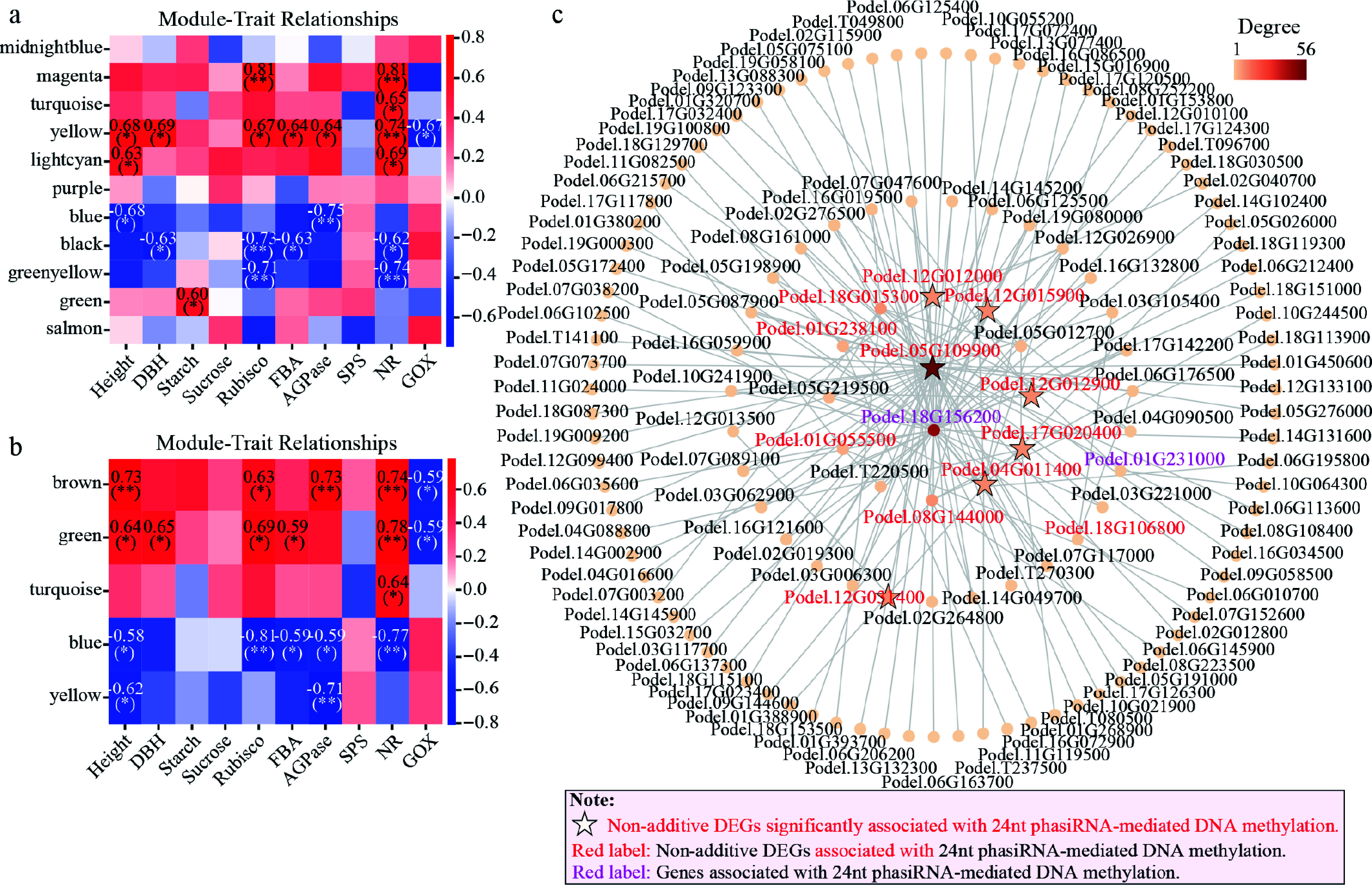
Weighted gene co-expression network analysis (WGCNA) of growth phenotype and biochemical traits correlated with DMGs. (a) Correlation between traits and modules in WGCNA for parental SMRs. (b) Correlation between traits and modules in WGCNA in parental DMRs. (c) Co-expression network diagram for key genes from the eight modules with genes correlated with 24-nt phasiRNA-associated DNA methylation.

The FPKM matrix of DMGs (16,517 and 5,250) between parents and hybrids in parent SMRs and DMRs was combined with growth traits and leaf physiological and biochemical traits for weighted gene co-expression network analysis (WGCNA)^[37]^. Four modules within both parental SMRs and DMRs were significantly correlated with traits (Fig. 7a, b), and module genes were differentially expressed between different hybrids (Supplementary Fig. S5a, S5b). Four genes (*Podel.12G012000*, *Podel.12G012900*, *Podel.12G031400*, and *Podel.01G238100*) were located in the significantly negatively correlated blue module of parental SMRs, while three genes (*Podel.12G012000*, *Podel.12G012900*, and *Podel.12G031400*) were in the significantly negatively correlated yellow module of parental DMRs (Supplementary Table S9). These genes were significantly correlated with 24-nt phasiRNA-associated CHG/CHH-Genebody-DMRs between hybrids and parents, accompanied by non-additive differential expression (Fig. 5c−e). The 24-nt phasiRNAs in different hybrids exhibited distinct association patterns with DNA methylation and gene expression: H2 mainly showed high 24-nt phasiRNA expression, Hyper-DMRs, and decreased gene expression, while L3 mainly showed low 24-nt phasiRNA expression, Hypo-DMRs, and increased gene expression (Fig. 6).

The top 50 genes with the highest connectivity from each of the eight trait-correlated modules were integrated with 29 genes correlated with 24-nt phasiRNA-associated DNA methylation, constructing gene relationships (*p* < 0.05, correlation coefficient ≥ 0.8). The co-expression network contained 132 genes potentially relevant to heterosis in *P. deltoides* (Fig. 7c), including 14 genes (12 non-additive genes) correlated with 24-nt phasiRNA-associated DNA methylation. Two hub genes, *Podel.05G109900* and *Podel.18G156200*, were linked to CHH-DMRs positively associated with 24-nt phasiRNA and showed inverse relationships between methylation and gene expression (Fig. 7c; Supplementary Table S10). H2 mainly showed lower 24-nt phasiRNAs expression, Hypo-DMRs, and increased gene expression, while L3 showed the opposite pattern. Additionally, 12 secondary key node genes were identified, with nine genes correlated with 24-nt phasiRNA-associated DNA methylation (*Podel.04G011400*, *Podel.17G020400*, *Podel.12G012000*, etc.) (Fig. 7c). The remaining three genes, *AUX/IAA* (*Podel.05G219500*), *PsbP* (*Podel.05G012700*), and *AGPase* (*Podel.T220500*), were heterosis-related genes^[47,37]^. The seven non-additive DEGs that were significantly correlated with 24-nt phasiRNAs(13)-associated DNA methylation mentioned above were all present in this co-expression network (Figs 6, 7c), suggesting that they may play a key role in *P. deltoides* heterosis. The 132 genes were mainly located in parental SMRs, mainly CG-genebody and CHG/CHH-promoter types (Supplementary Table S10). KEGG analysis showed significant enrichment in pathways such as SNARE interactions in vesicular transport, flavonoid biosynthesis, and carbon metabolism (Supplementary Fig. S5c; Supplementary Table S10). GO-BP enrichment analysis revealed these genes were involved in biological processes, including response to hormones, regulation of growth, photosynthesis, and cell wall biogenesis (Supplementary Fig. S5d; Supplementary Table S10). These findings suggest that genes correlated with 24-nt phasiRNA-associated DNA methylation, along with genes related to growth hormones, photosynthesis, and carbon metabolism, may collectively drive heterosis formation. These genes exhibit opposite expression patterns in hybrids and may play an important role in heterosis formation.

### qRT-PCR validation of key genes

To verify RNA-seq data, primers were designed for four key growth-related genes potentially modulated by 24-nt phasiRNA-associated DNA methylation, four methyltransferase genes, and two auxin signaling genes associated with DNA methylation (Supplementary Table S1). The qRT-PCR results showed that *Podel.06G125400*, *Podel.10G079100*, *Podel.01G029900*, *Podel.05G219500*, and *Podel.09G131600* were upregulated in H2 and downregulated in L3, while *Podel.12G012900* and *Podel.12G012000* were significantly downregulated in H2 and upregulated in L3 (Supplementary Fig. S6a). The qRT-PCR results aligned with the RNA-seq data (Supplementary Fig. S6b), validating the reproducibility and reliability of the transcriptomic data.

## Discussion

### DNA methylation and growth potential in *P. deltoides*

Our study reveals that F1 hybrids of *P. deltoides* exhibit reduced genome-wide DNA methylation, with differential CHG methylation patterns likely influencing hybrid growth potentials. Most methylation sites in F1 hybrids of *P. deltoides* were shared with parents, whereas only a small proportion were hybrid-specific (Fig. 1b), indicating that the hybrid methylome is largely inherited from the parents, as also reported in potato and *Cicer arietinum*^[12,14]^. Previous studies indicate that elevated DNA methylation contributes to biomass heterosis in *Arabidopsis* and *B. oleracea*^[19,48]^. However, the methylation pattern in poplar was distinct: the high-growth hybrid (H2) displayed intermediate methylation levels between those of the male parent (MP) and the female parent (FP), with a bias towards MP, whereas the low-growth hybrid (L3) exhibited higher methylation levels than both parents, with a bias towards FP. This methylation bias may lead to dominance or complete dominance of genes and traits, potentially driving heterosis^[49]^. We suggest that DNA methylation plays a distinct role in growth heterosis in *P. deltoides* compared to herbaceous plants, likely due to the highly heterozygous, long-lived, and stationary nature of woody plants, whose genome-wide methylation is more influenced by environmental factors.

At the sequence-context level, CHG and CHH methylation were especially dynamic. Previous studies have shown that parental CHG methylation divergence can contribute to epigenetic reprogramming and heterotic phenotypes in rice and *Arabidopsis* hybrids^[2,21]^. In *P. deltoides*, the number of Hyper- and Hypo-DMRs primarily involves CHG and CHH contexts. More Hypo-DMRs were found between H2 and parents, most of which were CHG-DMRs, suggesting that CHG methylation predominantly drove F1 hybrid variation. Furthermore, in CHH contexts, F1 hybrids primarily showed Hypo-DMRs relative to FP and Hyper-DMRs relative to MP, differences likely driven by parental sRNA variation^[50]^. These findings highlight the significant influence of parental epigenetic divergence on CHG and CHH methylation reprogramming in F1 hybrids.

### Hybrid-parent DMRs from parental SMRs likely contribute to poplar heterosis

Our results revealed that DMRs were predominantly distributed in promoter, genebody, and TE regions, which are essential for gene regulation and genomic stability^[20,24]^. Specifically, within parental DMRs, hybrid-parent DMRs in promoter and genebody regions often occurred where the female parent (FP) exhibited lower methylation than the male parent (MP)—a trend opposite to global methylation patterns. This suggests that localized parental methylation differences primarily drive F1 hybrid variation^[21]^. Studies on epigenetic hybrids in *Arabidopsis* have demonstrated that genetic differences between parents increase the likelihood of hybrid variation^[21]^. However, parental DMRs are not the primary regions of non-additive methylation changes in F1 hybrids^[48,51]^; our findings suggest that F1 hybrid-parent DMRs in *P. deltoides* primarily originate from parental SMRs. Thus, parental SMRs likely contribute to epigenetic variation and growth heterosis in hybrids.

We observed distinct patterns of promoter and genebody methylation-associated DMEGs between F1 hybrids with different growth potentials and parents. H2 predominantly exhibited positive correlations (e.g., hypermethylation accompanied by increased gene expression), while L3 showed primarily negative correlations. While classical models typically posit a global negative correlation between DNA methylation and transcription, our analysis reveals a highly context-dependent epigenetic landscape^[52]^. Promoter hypermethylation typically represses gene expression, but genebody hypermethylation—particularly in CG contexts—frequently positively correlates with actively transcribed genes, preventing cryptic transcription initiation and maintaining transcriptional fidelity^[53]^. These divergent localized epigenetic reprogramming strategies between H2 and L3 highlight the importance of their associated biological functions. These DMEGs are largely associated with defense mechanisms and starch and sucrose metabolism, critical for balancing growth and defense^[54]^. Similar to findings in *Cicer arietinum* and soybean hybrids^[12,55]^, DNA methylation modulated key genes involved in sucrose/starch synthesis, carbon fixation, and nitrogen metabolism in *P. deltoides*. These DMEGs predominantly exhibited high-parental dominant expression (HP) patterns in H2, whereas signal transduction and stress-responsive DMEGs were primarily upregulated in L3. The epigenetic divergence in these parent-dominant genes may promote heterosis by maintaining critical physiological processes in the high-growth hybrid^[12,56]^.

### 24-nt phasiRNA-associated CHG/CHH DNA methylation contributes to non-additive gene expression

The abundance of 24-nt sRNAs differed significantly between hybrids and parents, with non-additive expression potentially playing a role in heterosis formation. Previous studies have documented decreased 24-nt siRNA levels in hybrids exhibiting biomass heterosis, such as maize and *Arabidopsis*^[10,19,57]^; non-additive 24-nt phasiRNAs in *P. deltoides* F1 hybrids mainly exhibited negative dominance (Fig. 3d). Although parental differences in siRNA expression contribute to non-additive expression in *Arabidopsis* hybrids^[29]^, our findings indicate that differentially expressed parental 24-nt phasiRNAs are not the main source of non-additive expression in *P. deltoides* F1 hybrids.

Canonical 24-nt siRNA-associated RdDM depends on the Pol IV–RDR2–DCL3 pathway; however, loss of the RDR2 homolog or mutation of Pol IV does not eliminate the heterosis phenotype^[57]^, whereas 24-nt phasiRNAs produced via the Pol II–RDR6–DCL3/DCL5 pathway are linked to DNA methylation changes^[31]^. Therefore, although 24-nt phasiRNAs are a special class of secondary siRNAs, their biogenesis is relatively independent from the canonical RdDM pathway. Research on 24-nt phasiRNAs has primarily focused on reproductive development in rice, maize, and lychee (*Litchi chinensis*), proposing that 24-nt phasiRNAs may *cis*-regulate DNA methylation in maize^[34,58]^. Our study presents the first evidence that 24-nt phasiRNA abundance is positively correlated with methylation levels in tree species (Figs 5b, 6). This finding is consistent with 24-nt siRNA^[20]^.

Studies have reported that 24-nt siRNAs are generally positively associated with methylation in the corresponding regions and non-additive differential expression of genes involved in key growth- and development-related pathways, through the RdDM pathway, thus contributing to heterosis^[12,19]^. Similarly, we identified 13 24-nt phasiRNAs (five non-additive) that were significantly linked to hybrid-parent DMRs and seven non-additive DMEGs, with varying expression in different hybrids. These DMEGs may be key genes in the gene co-expression network associated with growth heterosis in *P. deltoides*, with important biological functions. For instance, the *NLP2/NLP8-like* gene (*Podel.17G020400*) is involved in coordinating nitrogen assimilation and carbon skeleton supply^[59]^; *PsbP-like* *protein 1* (*Podel.12G015900*) optimizes Photosystem II efficiency, directly enhancing photosynthetic capacity^[60]^; and *mTERF* (*Podel.04G011400*) is associated with chloroplast transcription and photosynthetic electron transport^[61]^. Furthermore, the *RRN3* gene (*RNA polymerase I-specific transcription initiation factor*, *Podel.05G109900*) initiates ribosomal RNA transcription; its upregulation represents a rate-limiting step for massive protein synthesis and cellular proliferation characteristic of robust plant vigor^[62]^. These growth-related genes, linked to 24-nt phasiRNA-associated CHG/CHH DMRs, were dominantly upregulated in H2 and displayed reverse epigenetic patterns between hybrids. Specifically, H2 mainly exhibited downregulated 24-nt phasiRNAs, hypomethylation, and increased gene expression, whereas 24-nt phasiRNAs upregulation, hypermethylation, and decreased gene expression were exhibited in L3 (Fig. 5c−e). Conversely, defense-related *CHS* and *LRR* genes (*Podel.12G012000*, *Podel.12G012900*, and *Podel.12G031400*), correlated with the downregulation of 24-nt phasiRNA and CHG/CHH hypomethylation, were dominantly upregulated in L3; whereas H2 showed a pattern of 24-nt phasiRNAs upregulation, CHG/CHH hypermethylation, and decreased gene expression (Fig. 5c−e).

Additionally, non-additive 24-nt phasiRNAs were positively associated with CHG Promoter-DMRs and negatively correlated with *NLP2/NLP8-like* expression (Fig. 6). In H2, downregulated 24-nt phasiRNAs corresponded to hypo-DMRs and high parental expression of *NLP2/NLP8-like*, whereas L3 exhibited elevated novel phasiRNA-431-3, hypermethylation, and lower gene expression. Meanwhile, non-additive 24-nt phasiRNAs were positively associated with CHH Promoter-DMRs and negatively correlated with *RRN3* expression. H2 exhibited the downregulation of 24-nt phasiRNAs, hypomethylation, and high parental expression of *RRN3*, whereas L3 showed elevated novel phasiRNA-431-3, hypermethylation, and lower gene expression. Therefore, parent-dominant gene expression, correlated with 24-nt phasiRNAs-associated CHG/CHH methylation, may promote growth heterosis in *P. deltoides*. As is well recognized, growth heterosis in *P. deltoides* involves highly complex molecular mechanisms. To further validate the general applicability of the above regulatory mechanism in poplar heterosis, we will incorporate additional independent hybrid lines with consistent growth traits and diverse hybrid combinations for follow-up experimental verification.

## Conclusions

This study preliminarily elucidates the critical role of 24-nt phasiRNAs in mediating DNA methylation and regulating gene expression in *Populus* hybrids. Our findings reveal that the abundance and expression profiles of 24-nt phasiRNAs differ significantly between hybrids and parents, which may contribute to the formation of growth heterosis. The distinct inheritance patterns identified, including conserved, additive, dominance, and overdominance expressions, underscore the complex genetic and epigenetic interactions driving heterosis. Moreover, the transgenerational non-additive expression of phasiRNAs suggests their potential as key regulatory elements for enhancing biomass in plant breeding. By constructing a 24-nt phasiRNAs-DNA methylation-non-additive DEGs network, we identified 13 candidate 24-nt phasiRNAs and seven key genes involved in heterosis. Notably, three non-additive 24-nt phasiRNAs were significantly negatively correlated with non-additive gene expression, via positively associating with local DNA methylation. These key genes may execute specific biological functions essential for hybrid vigor: *NLP2/NLP8-like* is involved in nitrogen assimilation, *RRN3* regulates transcriptional initiation, and *PsbP* enhances carbon metabolism. Collectively, this research provides valuable correlational insights into the epigenetic mechanisms driving heterosis in tree genetics and breeding.

## Data Availability

The raw sequencing data of Transcriptome (accession number CRA018573), whole genome bisulfite sequencing (accession number CRA022611), and small RNA sequencing (accession number CRA022610) in this paper have been deposited in the Genome Sequence Archive in National Genomics Data Center, and are available at the National Genomics Data Center, China National Center for Bioinformation (GSA: CRA018573, CRA022611, CRA022610) that are publicly accessible at https://ngdc.cncb.ac.cn/gsa.
